# 4-Amino-2-chloro­benzoic acid

**DOI:** 10.1107/S1600536811030728

**Published:** 2011-08-06

**Authors:** Muneeb Hayat Khan, Islam Ullah Khan, Mehmet Akkurt

**Affiliations:** a Materials Chemistry Laboratory, Department of Chemistry, GC University, Lahore 54000, Pakistan; b Department of Physics, Faculty of Sciences, Erciyes University, 38039 Kayseri, Turkey

## Abstract

The title compound, C_7_H_6_ClNO_2_, crystallizes with two roughly planar mol­ecules in the asymmetric unit (r.m.s. deviations = 0.073 and 0.074 Å). The amine H atoms of the two mol­ecules have opposite orientations. In the crystal, mol­ecules are linked into dimers by pairs of O—H⋯O hydrogen bonds, generating *R*_2_^2^(8) loops. N—H⋯N and N—H⋯Cl hydrogen bonds link the dimers into a three-dimensional network. The crystal studied was found to be a racemic twin.

## Related literature

For an isomer (2-amino-4-chloro­benzoic acid) of the title compound, see: Farag *et al.* (2011 ▶). For hydrogen-bond motifs, see: Bernstein *et al.* (1995 ▶).
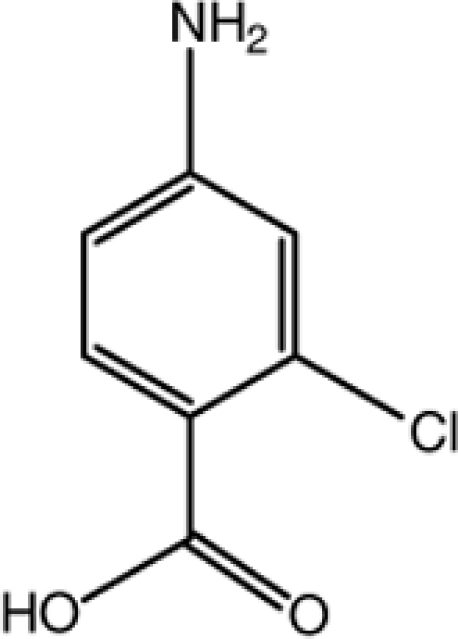


## Experimental

### 

#### Crystal data

C_7_H_6_ClNO_2_
*M* *_r_* = 171.58
Monoclinic, 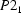

*a* = 3.9595 (2) Å
*b* = 22.6656 (11) Å
*c* = 8.0285 (4) Å
β = 104.257 (2)°
*V* = 698.32 (6) Å^3^
*Z* = 4
Mo *K*α radiation
μ = 0.49 mm^−1^
*T* = 296 K
0.28 × 0.13 × 0.12 mm

#### Data collection

Bruker APEXII CCD diffractometer
3009 measured reflections
2295 independent reflections
2197 reflections with *I* > 2σ(*I*)
*R*_int_ = 0.011

#### Refinement

*R*[*F* ^2^ > 2σ(*F* ^2^)] = 0.029
*wR*(*F* ^2^) = 0.077
*S* = 1.07
2295 reflections
218 parameters
7 restraints
H atoms treated by a mixture of independent and constrained refinement
Δρ_max_ = 0.21 e Å^−3^
Δρ_min_ = −0.23 e Å^−3^
Absolute structure: Flack (1983 ▶), 514 Freidel pairs
Flack parameter: 0.50 (6)

### 

Data collection: *APEX2* (Bruker, 2007 ▶); cell refinement: *SAINT* (Bruker, 2007 ▶); data reduction: *SAINT*; program(s) used to solve structure: *SIR97* (Altomare *et al.*, 1999 ▶); program(s) used to refine structure: *SHELXL97* (Sheldrick, 2008 ▶); molecular graphics: *ORTEP-3 for Windows* (Farrugia, 1997 ▶); software used to prepare material for publication: *WinGX* (Farrugia, 1999 ▶) and *PLATON* (Spek, 2009 ▶).

## Supplementary Material

Crystal structure: contains datablock(s) global, I. DOI: 10.1107/S1600536811030728/hb6334sup1.cif

Structure factors: contains datablock(s) I. DOI: 10.1107/S1600536811030728/hb6334Isup2.hkl

Supplementary material file. DOI: 10.1107/S1600536811030728/hb6334Isup3.cml

Additional supplementary materials:  crystallographic information; 3D view; checkCIF report

## Figures and Tables

**Table 1:** Hydrogen-bond geometry (Å, °)

*D*—H⋯*A*	*D*—H	H⋯*A*	*D*⋯*A*	*D*—H⋯*A*
O1—H*O*1⋯O4^i^	0.82 (3)	1.84 (3)	2.650 (3)	171 (3)
N1—H*N*2⋯N2	0.86 (3)	2.60 (3)	3.375 (4)	150 (3)
O3—H*O*3⋯O2^ii^	0.81 (3)	1.84 (3)	2.650 (3)	173 (3)
N2—H*N*3⋯Cl2^iii^	0.86 (3)	2.81 (3)	3.374 (2)	125 (2)
N2—H*N*4⋯N1^iv^	0.86 (3)	2.47 (3)	3.302 (4)	163 (2)

Symmetry codes: (i) 
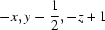
; (ii) 
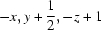
; (iii) 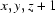; (iv) 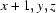.

**Fig. 1.**
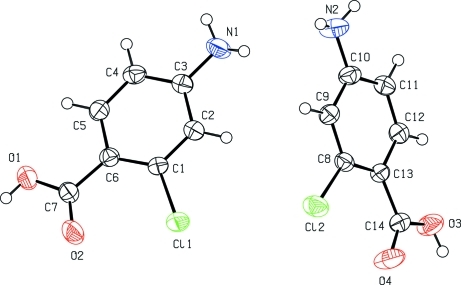
The molecule of the title compound, with displacement ellipsoids for non-H atoms drawn at the 50% probability level.

**Fig. 2.**
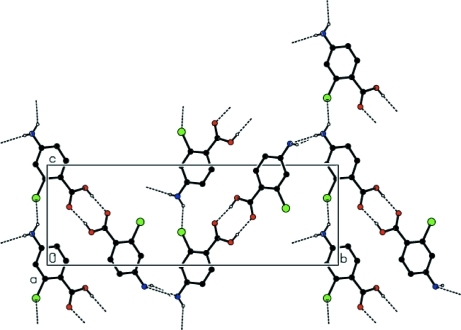
Partial view of the dimers by two O—H···O hydrogen bonds and the packing in the crystal. Hydrogen atoms that not involved in the hydrogen-bonding (dashed lines) have been omitted for clarity.

## supplementary crystallographic information

### Comment

The title compound, (I), is used as a starting material for the synthesis of various sulphonamides.

As shown in Fig. 1, the asymmetric unit of the title compound contains two independent molecules. Both have the bond lengths and angles as expected for a molecule of this kind (Farag *et al.*, 2011).

The amine H atoms of the two molecules have opposite orientations. In the crystal, the molecules form dimers *via* intermolecular O—H···O hydrogen bonds, forming a graph-set motif *R*^2^_2_(8) (Bernstein *et al.*, 1995; Table 1, Fig. 2). Furthermore, C—H···O, N—H···N and N—H···Cl interactions stabilize the crystal structure.

### Experimental

To a 100-ml round bottom flask equipped with a reflux condenser, was placed 0.5 g (2.486 mmol) of 2-chloro-4-amino benzoic acid and 0.447 g m of granulated tin.  Then, 30 ml of concentrated HCl in was added in six intervals (5 ml each time with cooling in ice). The reaction is highly exothermic and the reaction mixture is keept under control by keeping it in ice water. When all the HCl was added and the temprature of the reaction mixture was stable, the round bottom flask was placed on water bath for 90 min under reflux.

TLC check after 90 min showed completion of reaction. Reaction mixtures was treated with 60% NaOH solution followed by the addition of NaCl solution and extration with di-ethyl ether.

Diethyl ether was evaporated on rotary evaporator and reddish brown precipates of the required product were obtained. This was recrystallized in methanol to yield reddish brown prisms of (I).

### Refinement

The H atoms of the NH_2_ and OH groups in the title compound were located in a difference map and refined with the distance restraint N—–H = 0.86 (1) and O—–H = 0.82 (1) Å; their *U*_iso_ values were constrained to be 1.5*U*_eq_ of the carrier atom for hydroxyl H atoms and 1.2*U*_eq_ for amine H atoms. The remaining aromatic H atoms were positioned geometrically with C—H = 0.93 Å, and refined using a riding model, with *U*_iso_(H) = 1.2*U*_eq_(C). The crystal studied was a racemic twin [Flack parameter = 0.50 (6)].

### Crystal data

**Table:**

C_7_H_6_ClNO_2_	*F*(000) = 352
*M**_r_* = 171.58	*D*_x_ = 1.632 Mg m^−^^3^
Monoclinic, *P*2_1_	Mo *K*α radiation, λ = 0.71073 Å
Hall symbol: P 2yb	Cell parameters from 3211 reflections
*a* = 3.9595 (2) Å	θ = 2.8–28.3°
*b* = 22.6656 (11) Å	µ = 0.49 mm^−^^1^
*c* = 8.0285 (4) Å	*T* = 296 K
β = 104.257 (2)°	Prism, reddish brown
*V* = 698.32 (6) Å^3^	0.28 × 0.13 × 0.12 mm
*Z* = 4	

### Data collection

**Table:**

Bruker APEXII CCD diffractometer	2197 reflections with *I* > 2σ(*I*)
Radiation source: sealed tube	*R*_int_ = 0.011
graphite	θ_max_ = 28.3°, θ_min_ = 2.8°
φ and ω scans	*h* = −5→5
3009 measured reflections	*k* = −18→30
2295 independent reflections	*l* = −10→10

### Refinement

**Table:**

Refinement on *F*^2^	Secondary atom site location: difference Fourier map
Least-squares matrix: full	Hydrogen site location: inferred from neighbouring sites
*R*[*F*^2^ > 2σ(*F*^2^)] = 0.029	H atoms treated by a mixture of independent and constrained refinement
*wR*(*F*^2^) = 0.077	*w* = 1/[σ^2^(*F*_o_^2^) + (0.0454*P*)^2^ + 0.092*P*] where *P* = (*F*_o_^2^ + 2*F*_c_^2^)/3
*S* = 1.07	(Δ/σ)_max_ < 0.001
2295 reflections	Δρ_max_ = 0.21 e Å^−^^3^
218 parameters	Δρ_min_ = −0.23 e Å^−^^3^
7 restraints	Absolute structure: Flack (1983), 514 Freidel pairs
Primary atom site location: structure-invariant direct methods	Flack parameter: 0.50 (6)

### Special details

**Table:**

Geometry. Bond distances, angles *etc*. have been calculated using the rounded fractional coordinates. All su's are estimated from the variances of the (full) variance-covariance matrix. The cell e.s.d.'s are taken into account in the estimation of distances, angles and torsion angles
Refinement. Refinement on *F*^2^ for ALL reflections except those flagged by the user for potential systematic errors. Weighted *R*-factors *wR* and all goodnesses of fit *S* are based on *F*^2^, conventional *R*-factors *R* are based on *F*, with *F* set to zero for negative *F*^2^. The observed criterion of *F*^2^ > σ(*F*^2^) is used only for calculating -*R*-factor-obs *etc*. and is not relevant to the choice of reflections for refinement. *R*-factors based on *F*^2^ are statistically about twice as large as those based on *F*, and *R*-factors based on ALL data will be even larger.

### Fractional atomic coordinates and isotropic or equivalent isotropic displacement parameters (Å^2^)

**Table:**

	*x*	*y*	*z*	*U*_iso_*/*U*_eq_	
Cl1	0.23950 (15)	0.81998 (3)	0.56801 (6)	0.0427 (2)	
O1	−0.2726 (5)	0.65233 (9)	0.6710 (2)	0.0502 (6)	
O2	−0.0913 (6)	0.70711 (10)	0.4820 (2)	0.0577 (7)	
N1	0.5447 (5)	0.83421 (11)	1.2139 (3)	0.0431 (7)	
C1	0.2193 (5)	0.78948 (10)	0.7631 (3)	0.0277 (5)	
C2	0.3803 (5)	0.82200 (12)	0.9065 (3)	0.0316 (5)	
C3	0.3834 (5)	0.80158 (10)	1.0704 (3)	0.0306 (6)	
C4	0.2106 (6)	0.74934 (11)	1.0871 (3)	0.0349 (6)	
C5	0.0515 (6)	0.71758 (11)	0.9432 (3)	0.0338 (6)	
C6	0.0508 (5)	0.73617 (10)	0.7767 (3)	0.0294 (6)	
C7	−0.1085 (5)	0.69776 (11)	0.6301 (3)	0.0341 (6)	
Cl2	0.80948 (15)	0.96180 (3)	0.66956 (7)	0.0442 (2)	
O3	0.3027 (6)	1.13060 (8)	0.7718 (2)	0.0511 (6)	
O4	0.4834 (7)	1.07555 (10)	0.5823 (2)	0.0581 (7)	
N2	1.1098 (6)	0.94780 (12)	1.3131 (3)	0.0491 (8)	
C8	0.7883 (5)	0.99282 (10)	0.8643 (3)	0.0293 (5)	
C9	0.9466 (5)	0.96044 (12)	1.0074 (3)	0.0321 (5)	
C10	0.9516 (5)	0.98080 (11)	1.1714 (3)	0.0349 (6)	
C11	0.7808 (6)	1.03355 (12)	1.1881 (3)	0.0356 (6)	
C12	0.6218 (6)	1.06497 (11)	1.0437 (3)	0.0347 (6)	
C13	0.6238 (5)	1.04643 (10)	0.8776 (3)	0.0295 (6)	
C14	0.4640 (5)	1.08498 (11)	0.7309 (3)	0.0336 (6)	
HO1	−0.335 (8)	0.6315 (13)	0.585 (3)	0.0640*	
H2	0.48700	0.85770	0.89330	0.0380*	
HN1	0.607 (7)	0.8126 (13)	1.303 (3)	0.0510*	
HN2	0.707 (6)	0.8583 (11)	1.203 (4)	0.0510*	
H4	0.20270	0.73590	1.19550	0.0420*	
H5	−0.05990	0.68240	0.95690	0.0410*	
HO3	0.220 (8)	1.1532 (14)	0.694 (4)	0.0660*	
HN3	1.163 (7)	0.9675 (13)	1.407 (3)	0.0530*	
HN4	1.262 (6)	0.9232 (11)	1.296 (4)	0.0530*	
H9	1.05120	0.92460	0.99420	0.0390*	
H11	0.77480	1.04740	1.29640	0.0430*	
H12	0.50820	1.09990	1.05690	0.0420*	

### Atomic displacement parameters (Å^2^)

**Table:**

	*U*^11^	*U*^22^	*U*^33^	*U*^12^	*U*^13^	*U*^23^
Cl1	0.0554 (3)	0.0449 (3)	0.0274 (2)	−0.0095 (3)	0.0097 (2)	0.0044 (2)
O1	0.0723 (11)	0.0413 (11)	0.0365 (9)	−0.0205 (9)	0.0123 (8)	−0.0066 (8)
O2	0.0901 (14)	0.0528 (12)	0.0312 (8)	−0.0299 (11)	0.0170 (9)	−0.0109 (9)
N1	0.0473 (10)	0.0514 (15)	0.0276 (9)	−0.0042 (9)	0.0033 (8)	−0.0061 (9)
C1	0.0280 (8)	0.0302 (11)	0.0252 (9)	0.0023 (8)	0.0073 (7)	0.0022 (8)
C2	0.0313 (8)	0.0335 (11)	0.0290 (9)	0.0005 (9)	0.0058 (7)	−0.0018 (10)
C3	0.0292 (8)	0.0340 (12)	0.0281 (9)	0.0038 (8)	0.0061 (7)	−0.0020 (8)
C4	0.0407 (10)	0.0384 (13)	0.0267 (10)	0.0060 (9)	0.0103 (9)	0.0025 (9)
C5	0.0384 (10)	0.0301 (12)	0.0332 (11)	−0.0001 (9)	0.0097 (8)	0.0019 (9)
C6	0.0316 (9)	0.0288 (11)	0.0274 (10)	0.0026 (8)	0.0068 (7)	0.0004 (8)
C7	0.0355 (9)	0.0323 (12)	0.0328 (11)	−0.0013 (8)	0.0053 (8)	−0.0034 (9)
Cl2	0.0540 (3)	0.0475 (3)	0.0305 (3)	0.0080 (3)	0.0091 (2)	−0.0060 (3)
O3	0.0750 (12)	0.0388 (11)	0.0384 (9)	0.0208 (10)	0.0121 (8)	0.0072 (9)
O4	0.0890 (14)	0.0535 (12)	0.0324 (9)	0.0303 (11)	0.0163 (9)	0.0112 (9)
N2	0.0545 (11)	0.0603 (17)	0.0320 (10)	0.0120 (11)	0.0095 (9)	0.0132 (11)
C8	0.0304 (8)	0.0325 (11)	0.0253 (9)	−0.0041 (8)	0.0073 (7)	−0.0026 (9)
C9	0.0319 (8)	0.0304 (10)	0.0335 (9)	0.0030 (9)	0.0072 (7)	0.0026 (10)
C10	0.0322 (9)	0.0419 (13)	0.0298 (10)	−0.0042 (9)	0.0062 (8)	0.0083 (9)
C11	0.0419 (10)	0.0379 (13)	0.0266 (10)	−0.0022 (9)	0.0077 (9)	−0.0037 (9)
C12	0.0421 (10)	0.0308 (11)	0.0314 (10)	−0.0001 (9)	0.0094 (8)	−0.0029 (9)
C13	0.0310 (9)	0.0290 (11)	0.0278 (10)	−0.0019 (8)	0.0057 (8)	0.0033 (8)
C14	0.0391 (10)	0.0322 (12)	0.0291 (10)	0.0004 (9)	0.0078 (8)	0.0039 (9)

### Geometric parameters (Å, °)

**Table:**

Cl1—C1	1.732 (2)	C3—C4	1.390 (3)
Cl2—C8	1.735 (2)	C4—C5	1.375 (3)
O1—C7	1.302 (3)	C5—C6	1.401 (3)
O2—C7	1.226 (3)	C6—C7	1.475 (3)
O1—HO1	0.82 (3)	C2—H2	0.9300
O3—C14	1.299 (3)	C4—H4	0.9300
O4—C14	1.233 (3)	C5—H5	0.9300
O3—HO3	0.81 (3)	C8—C9	1.377 (3)
N1—C3	1.386 (3)	C8—C13	1.395 (3)
N1—HN1	0.85 (3)	C9—C10	1.391 (3)
N1—HN2	0.86 (3)	C10—C11	1.396 (4)
N2—C10	1.377 (3)	C11—C12	1.374 (3)
N2—HN4	0.86 (3)	C12—C13	1.400 (3)
N2—HN3	0.86 (3)	C13—C14	1.478 (3)
C1—C6	1.398 (3)	C9—H9	0.9300
C1—C2	1.383 (3)	C11—H11	0.9300
C2—C3	1.392 (3)	C12—H12	0.9300
			
C7—O1—HO1	108 (2)	C5—C4—H4	120.00
C14—O3—HO3	116 (2)	C3—C4—H4	120.00
C3—N1—HN1	111.7 (19)	C4—C5—H5	119.00
HN1—N1—HN2	112 (3)	C6—C5—H5	119.00
C3—N1—HN2	117 (2)	C9—C8—C13	121.7 (2)
C10—N2—HN4	115 (2)	Cl2—C8—C9	115.01 (18)
HN3—N2—HN4	117 (3)	Cl2—C8—C13	123.30 (18)
C10—N2—HN3	114.0 (19)	C8—C9—C10	120.7 (2)
Cl1—C1—C2	115.19 (18)	N2—C10—C11	121.2 (2)
Cl1—C1—C6	123.03 (18)	C9—C10—C11	118.7 (2)
C2—C1—C6	121.8 (2)	N2—C10—C9	120.0 (2)
C1—C2—C3	120.3 (2)	C10—C11—C12	119.7 (2)
C2—C3—C4	119.0 (2)	C11—C12—C13	122.6 (2)
N1—C3—C4	120.8 (2)	C8—C13—C12	116.5 (2)
N1—C3—C2	120.2 (2)	C8—C13—C14	124.8 (2)
C3—C4—C5	120.0 (2)	C12—C13—C14	118.7 (2)
C4—C5—C6	122.5 (2)	O3—C14—C13	114.2 (2)
C5—C6—C7	118.9 (2)	O4—C14—C13	123.5 (2)
C1—C6—C7	124.5 (2)	O3—C14—O4	122.3 (2)
C1—C6—C5	116.5 (2)	C8—C9—H9	120.00
O2—C7—C6	123.8 (2)	C10—C9—H9	120.00
O1—C7—C6	114.0 (2)	C10—C11—H11	120.00
O1—C7—O2	122.1 (2)	C12—C11—H11	120.00
C1—C2—H2	120.00	C11—C12—H12	119.00
C3—C2—H2	120.00	C13—C12—H12	119.00
			
Cl1—C1—C2—C3	−179.44 (17)	Cl2—C8—C9—C10	179.22 (17)
C6—C1—C2—C3	1.1 (3)	C13—C8—C9—C10	−0.9 (3)
Cl1—C1—C6—C5	−179.00 (17)	Cl2—C8—C13—C12	178.57 (17)
Cl1—C1—C6—C7	4.2 (3)	Cl2—C8—C13—C14	−3.3 (3)
C2—C1—C6—C5	0.4 (3)	C9—C8—C13—C12	−1.3 (3)
C2—C1—C6—C7	−176.4 (2)	C9—C8—C13—C14	176.8 (2)
C1—C2—C3—N1	−180.0 (2)	C8—C9—C10—N2	179.8 (2)
C1—C2—C3—C4	−2.5 (3)	C8—C9—C10—C11	2.6 (3)
N1—C3—C4—C5	179.9 (2)	N2—C10—C11—C12	−179.2 (2)
C2—C3—C4—C5	2.5 (3)	C9—C10—C11—C12	−2.0 (3)
C3—C4—C5—C6	−1.0 (4)	C10—C11—C12—C13	−0.2 (4)
C4—C5—C6—C1	−0.5 (3)	C11—C12—C13—C8	1.9 (3)
C4—C5—C6—C7	176.5 (2)	C11—C12—C13—C14	−176.4 (2)
C1—C6—C7—O1	−175.4 (2)	C8—C13—C14—O3	175.1 (2)
C1—C6—C7—O2	4.4 (4)	C8—C13—C14—O4	−5.7 (4)
C5—C6—C7—O1	7.8 (3)	C12—C13—C14—O3	−6.8 (3)
C5—C6—C7—O2	−172.3 (2)	C12—C13—C14—O4	172.4 (2)

### Hydrogen-bond geometry (Å, °)

**Table:**

*D*—H···*A*	*D*—H	H···*A*	*D*···*A*	*D*—H···*A*
O1—HO1···O4^i^	0.82 (3)	1.84 (3)	2.650 (3)	171 (3)
N1—HN2···N2	0.86 (3)	2.60 (3)	3.375 (4)	150 (3)
O3—HO3···O2^ii^	0.81 (3)	1.84 (3)	2.650 (3)	173 (3)
N2—HN3···Cl2^iii^	0.86 (3)	2.81 (3)	3.374 (2)	125 (2)
N2—HN4···N1^iv^	0.86 (3)	2.47 (3)	3.302 (4)	163 (2)

Symmetry codes: (i) −*x*, *y*−1/2, −*z*+1; (ii) −*x*, *y*+1/2, −*z*+1; (iii) *x*, *y*, *z*+1; (iv) *x*+1, *y*, *z*.
